# Overlap and distinction between fear of cancer recurrence and health anxiety in long-term colorectal cancer survivors

**DOI:** 10.1007/s11764-026-02058-6

**Published:** 2026-06-18

**Authors:** Katrine Ingeman, Johanne D. Lyhne, Per Fink, Allan “Ben” Smith, Lisbeth Frostholm, Tina Birgitte Wisbech Carstensen

**Affiliations:** 1 Department of Functional Disorders, Aarhus University Hospital, Palle Juul-Jensens Boulevard 11, Aarhus N, 8200 Denmark; 2 Department of Clinical Oncology, University Hospital of Southern Denmark, Beriderbakken 4, Vejle, 7100 Denmark; 3 Department of Clinical Medicine, Aarhus University, Palle Juul-Jensens Boulevard 11, Aarhus N, 8200 Denmark; 4 The Daffodil Centre, The University of Sydney, A Joint Venture With Cancer Council NSW, Sydney, NSW Australia; 5 South West Sydney Clinical Campuses, Faculty of Medicine and Health, University of New South Wales (UNSW Sydney), Liverpool, Australia

**Keywords:** Fear of cancer recurrence, Psycho-oncology, Health anxiety, Cancer survivorship

## Abstract

**Background:**

Fear of cancer recurrence (FCR) and health anxiety (HA) share central features such as illness-related worries and hypervigilance toward bodily sensations, yet the conceptual boundaries between them remain debated. The present study investigated psychological clinical characteristics and treatment outcomes in people with FCR and HA.

**Methods:**

Data were drawn from a national cohort and a randomized controlled trial evaluating a therapist-guided internet-delivered intervention for FCR (TG-iConquerFear). Participants (≥ 5 years post-diagnosis of colorectal cancer, no recurrence) completed pre-intervention assessments of HA and FCR severity, along with self-report measures of FCR, illness worries, and psychological distress.

**Results:**

Among 298 interviewed participants, 38 met the criteria for clinical FCR only, 44 for both clinical FCR and HA, and 5 for HA only. Participants with comorbid HA more frequently reported illness worries predating cancer diagnosis (44%) compared to participants with FCR only (5.3%). For participants with FCR only, 42.1% reported worries limited to colorectal cancer, while this was the case for only 2.3% of participants with both FCR and HA. The latter group also had higher levels of depression (*P* = 0.005), anxiety (*P* < 0.001), and emotional distress (*P* = 0.005). Based on descriptive data, individuals with FCR only appeared to show a larger reduction in FCR symptoms following TG-iConquerFear than those with comorbid HA.

**Conclusion:**

FCR and HA are distinct, yet highly overlapping conditions among long-term colorectal cancer survivors. Comorbid HA is associated with more severe emotional distress.

**Implications for Cancer Survivors:**

Survivors will benefit from destigmatization of HA and correct distinction between FCR and HA to receive effective intervention.

**Supplementary information:**

The online version contains supplementary material available at https://doi.org/10.1007/s11764-026-02058-6.

## Introduction

There is a rising concern for the psychological well-being of cancer survivors [1, 2]. Fear of cancer recurrence (FCR) has been identified as one of the most urgent unaddressed psychosocial needs for survivors [3–5]. FCR is defined as *fear*,* worry*, or* concern about cancer returning or progressing* [6], and moderate–severe levels are experienced by 58.8% of all cancer survivors [7]. For some, FCR is not just transient but becomes a clinical problem which is estimated to occur in 19.2% across survivors and 5.3% of long-term colorectal cancer survivors, defined by Fear of Cancer Recurrence Inventory – short form (FCRI-SF) scores ≥ 22 [7–9]. In a Delphi process in 2020, experts in psycho-oncology agreed that clinical FCR is characterized by (1) *high levels of preoccupation with recurrence*, (2) *high levels of worry*, (3) *being persistent (*> *3 months)*, and (4) *hypervigilance to bodily symptoms* [10]. Furthermore, although FCR is not yet a formal clinical diagnosis, it is well-established that FCR is associated with mental disorders like depression, anxiety, and health anxiety (HA) specifically [1, 9].

The term HA is used by researchers and clinicians to describe worries about illness [11]. HA may be used as an umbrella term covering a continuum of illness worries [12] or synonymously with terms used for psychiatric diagnosis. In the diagnostic classification systems, HA is classified as hypochondriasis (health anxiety disorder) in the ICD-11 [13], and in the DSM-5, it is classified as illness anxiety disorder or somatic symptom disorder depending on the presentation of somatic symptoms [14]. Severe HA is characterized by high levels of preoccupation and high levels of persistent worries about illness [15]. A core feature is hypervigilance to bodily symptoms together with the conviction or fear that bodily sensations are caused by a serious disease, including but not limited to cancer [16]. Fink and colleagues have proposed diagnostic criteria for HA based on empirical data defining obsessive illness rumination as the core symptom characteristic and five accompanying symptoms covering preoccupation with health concerns or bodily sensations, suggestibility, excessive fascination with medical information, fear of medication, or fear of contamination [15] (see Textbox S1 in supplementary material). Both Fink’s criteria and the criteria for illness anxiety disorder include the requirement that, if a medical condition is present, the preoccupation and worry must be clearly excessive or disproportionate.

There is an ongoing debate about how to conceptualize HA in relation to FCR, and several researchers have addressed the theoretical and clinical overlaps between HA and FCR [11, 17–20]. When used as an umbrella term, HA has been suggested to include FCR [11, 19, 21]. Within the area of cancer survivorship, researchers and clinicians generally agree that the constructs should be separated in order to address and treat them effectively in clinical settings [6, 10, 18, 22]. Diagnosing HA in cancer survivors has raised concerns about the risk of over-diagnosing individuals as mentally ill, when their fear could be considered realistic given their condition [23]. Others argue that when worries develop into persistent rumination with an impact on daily functioning and quality of life, FCR becomes clinical and may be designated as HA [21, 24]. To the best of our knowledge, only a few studies have investigated FCR specifically in relation to HA [25–28]. One study found that 43% of breast cancer survivors who had been cancer free for at least a year had clinical levels of both FCR and HA according to validated self-report questionnaires [27]. Another study found that 16% of thyroid cancer survivors had clinically significant scores on HA measures 3–9 years after diagnosis [26]. To the best of our knowledge, no previous study has investigated FCR and HA using clinical diagnostic interviews. Conceptualizing HA in relation to FCR is important to destigmatize psychological late effects after cancer, specify clinical characteristics for better assessment, and secure effective treatments for both.

The present study is part of a series of studies investigating psychosocial late effects and internet-delivered treatment of FCR in long-term colorectal cancer survivors who are cancer free [29]. In one of the studies, we found a large overlap between FCR and HA on questionnaire level [9]. A sample of long-term cancer-free survivors provides a unique opportunity to compare FCR and HA, as the risk of recurrence is low, and the acute phase of survivorship has passed which increases the likelihood that persistent or excessive fear may reflect processes characteristic of HA like disproportionate threat appraisal or persistent illness-related rumination.

### Aim

In this study, we aim to (1) investigate psychological clinical characteristics in participants with clinical FCR and in participants with both FCR and HA as defined by Fink et al. [15] and (2) compare treatment outcomes (level of FCR and illness worries) for participants with clinical FCR only, versus clinical FCR and HA combined at 3-month follow-up.

## Method

### Study design and data sources

The data presented in this study derives from a large national study on late effects in long-term colorectal cancer survivors in Denmark (≥ 5 years since cancer diagnosis with no recurrence and no residual disease), including a randomized controlled trial (RCT) of a therapist-guided internet-delivered intervention for FCR (see protocol: Lyhne et al. [29]). The present paper reports secondary exploratory analyses of questionnaire data and clinical assessments of FCR and HA from the RCT of the internet-delivered treatment of FCR [30].

### Study sample

All colorectal cancer survivors in Denmark, located through the Danish Colorectal Cancer Database (DCCG) [31], were invited to participate if they fulfilled the following inclusion criteria: (a) above 18 years, (b) diagnosed with colorectal cancer between 2014 and 2018, and (c) treated with curative intent. The questionnaire was distributed electronically from REDCap [32, 33] between May 2023 and April 2024 to a digital citizen mailbox used for communication between citizens and public authorities in Denmark.

Participants were divided into two groups as part of the recruitment for the RCT:

1. Individuals with FCRI-SF scores ≥ 22 (i.e., above the established cutoff for clinically significant FCR [8, 34] and the inclusion criteria for the RCT) were offered a diagnostic assessment as part of the RCT inclusion process. These assessments were conducted within 2 weeks of survey completion to facilitate timely enrolment.
2. A random subset of individuals with FCRI-SF scores < 22 was invited to undergo a diagnostic assessment to evaluate the robustness of the cutoff [35] and to identify clinically relevant cases not captured by the threshold. These participants were not eligible for inclusion in the RCT; instead, they were randomly selected by clinicians from the pool of consenting respondents who had agreed to be contacted for a diagnostic assessment interview, irrespective of the time elapsed since questionnaire completion.

In both groups, assessors had access to participants’ questionnaire scores in advance as part of their preparation for the diagnostic assessment.

Inclusion criteria for the assessment were as follows: (a) filled in the FCRI-SF questionnaire, (b) had not had an incidence of cancer recurrence or new cancer, and (c) gave consent to a diagnostic assessment. At the assessment, participants were excluded if they did not speak Danish, had a severe psychiatric diagnosis other than HA, or were not interested in the RCT (for relevant participants).

### Intervention and RCT

Participants in the RCT were randomized to either internet-delivered treatment of FCR or augmented treatment as usual. The latter included the assessment and referral to a website with a non-guided, publicly available E-learning program in cancer rehabilitation. The intervention was a therapist-guided (TG) version of iConquerFear, which was originally a self-guided digital intervention derived from the face-to-face therapist-delivered ConquerFear [29, 36, 37]. The TG-iConquerFear was designed to reduce FCR and comprised of six modules delivered over 10 weeks containing educational text, interactive exercises, and short videos focusing on attention training, increasing metacognitive awareness, acceptance strategies, detached mindfulness, promotion of appropriate screening behavior, and values-based goal setting. The participants received weekly written feedback from an experienced therapist delivered asynchronously via an embedded text-messaging system.

### Investigated psychological clinical characteristics

In this study, the characteristics investigated from the assessment were onset of illness worries, subject of illness worries, number of late effects, and psychiatric comorbidity. The characteristics investigated from the questionnaires were subscales on the FCRI, symptoms of emotional distress, anxiety, and depression measured using the Symptom Checklist and illness worries measured using the Whiteley-6-R (see below).

### Measures

#### Demographics

Demographic information regarding age, sex, cancer type, and treatment were extracted from the DCCG registry. Participants self-reported additional demographic (education, marital status, occupational status, children) and clinical information (treatment course).

#### Clinical assessment

Clinical assessments were conducted over the phone by one of three psychologists with previous (5–20 years) clinical experience with assessment and treatment of HA. The psychologists supervised each other, and the first six interviews were conducted together in pairs of two.

The psychologists were trained in and used the Research Interview for Functional somatic Disorders (RIFD) [38]—a modified version of Schedules for Clinical Assessment in Neuropsychiatry (SCAN) [39]—to assess HA, depression, panic disorder, social anxiety, generalized anxiety, and obsessive-compulsive disorder and to screen for psychotic experiences. The RIFD was supplemented with questions related to cancer course and late effects after cancer. Psychologists assessed if illness worries started before or after cancer diagnosis, if the worries were exacerbated by cancer diagnosis, and if the illness worries were limited to colorectal cancer or extended to other illnesses.

##### Assessment of HA

HA was assessed using the diagnostic research criteria established by Fink et al. [15]. The core symptom for HA is illness rumination [15] which needs to be present together with at least one associated symptom to fulfill diagnostic criteria (see supplementary material for diagnostic criteria, Textbox S1). To identify ruminations, the psychologists specifically assessed whether the worries were obsessive and interfered with daily life including emotional and overall functional impact (work, family, leisure).

##### Assessment of FCR

The assessment of FCR did not adhere to specific criteria for clinical FCR as the original RCT study did not rely on this. Psychologists rated the level of FCR based on the assessment of illness-related worries, persistence of worries, and preoccupation with and hypervigilance to bodily symptoms which reflect the consensus-based criteria for clinical FCR [10]. In addition, control or avoidance behavior and the participants’ answer to the following question, “How much have the mentioned concerns about fear of recurrence overall disrupted your usual daily activities?”, informed the rating. The psychologists rated either *no impact*, *little impact or discomfort*, *moderate impact or discomfort*, or *severe and invalidating impact or discomfort*. The psychologist assessed the overall functional impact (emotional, work, family, leisure) during the last 6 months. No or little impact corresponded to non-clinical FCR while moderate to severe impact corresponded to clinical FCR.

##### Late effects other than FCR

A psychologist registered the presence of known late effects after colorectal cancer by asking participants if they experienced any of the following symptoms: abdominal pain, diarrhea/frequent loose stool, difficulty emptying bowels, involuntary passage of gas/loose stool, defecation urgency, involuntary urination, bleeding from the bladder, frequent urination, difficulty emptying the bladder, bladder pain, sexual dysfunction, peripheral neuropathy, fatigue, memory issues, or difficulty concentrating.

#### Questionnaire data

##### Fear of cancer recurrence inventory

The FCRI is a validated 42-item self-report measure used to assess FCR [8] and was the primary outcome measure in the RCT study [30]. It contains seven subscales (triggers, severity, psychological distress, functioning impairment, insight, reassurance, and coping strategies) which are all answered on a 5-point rating scale with higher scores indicating more severe symptoms (total score range 0–168) [40]. The FCRI has shown good test–retest reliability and convergent validity with other measures of FCR [8]. Internal consistency/reliability of the FCRI in the present study was good as measured using Cronbach’s alpha (*r *= 0.95).

The nine-item severity subscale is also used as the short form of the FCRI (FCRI_SF) which is answered on a 5-point scale (range 0–36, with higher scores indicating more fear). The FCRI-SF has also shown good test–retest reliability and convergent validity with other measures of FCR [8] and has been validated in Danish [35]. In the present study, the internal reliability of the FCRI-SF was good as measured using Cronbach’s alpha (*r* = 0.93).

##### Illness worries

The Whiteley Index 6-item revised version (Whiteley-6-R) was used to measure illness worries and preoccupation with fear of suffering from a serious illness [41, 42]. Questions are rated from 0, “not at all,” to 4, “a lot,” and a sum score is calculated from the six items (range 0–24). The Whiteley-6-R has shown to have good external validity and high reliability [41]. In the present study, the internal reliability was good measured using Cronbach’s alpha (*r* = 0.93).

##### Symptom checklist 

The SCL-8 [43], SCL-ANX4, and SCL-DEP6 [44] are subscales from the Symptom Checklist-90-Revised [45] and measure emotional distress, anxiety, and depression. It has shown satisfactory sensitivity and specificity in detecting depression and anxiety and emotional distress in relation to emotional psychiatric disorders [43, 44]. It is scored from 0, “Not at all,” to 4 “Extremely.” Internal validity in the present study was good for all three subscales: SCL-ANX4 = 0.89, SCL-8 = 0.93, and SCL-DEP6 = 0.87.

### Statistics

Analyses were conducted using Stata 18 [46]. Descriptive statistics were calculated for participants’ demographic and clinical information. Continuous variables were summarized as means and standard deviations (SD) for normally distributed data and as medians and ranges for non-normally distributed data. Categorical variables were presented as frequencies and percentages and compared using chi^2^. Comparisons of continuous variables were performed using independent *t*-tests for parametric data and Mann–Whitney *U* tests for non-parametric data. Significance levels were adjusted using Bonferroni correction. Results from assessments and treatment outcomes were reported using descriptive statistics only due to the small sample sizes.

### Ethics

All participants provided electronic and verbal consent to participate in the study. The study was approved by The Regional Committee on Health Research Ethics for Southern Denmark (Project-ID S-20190061) and was in agreement with the Declaration of Helsinki.

## Results

### Participants

**Fig. 1:**
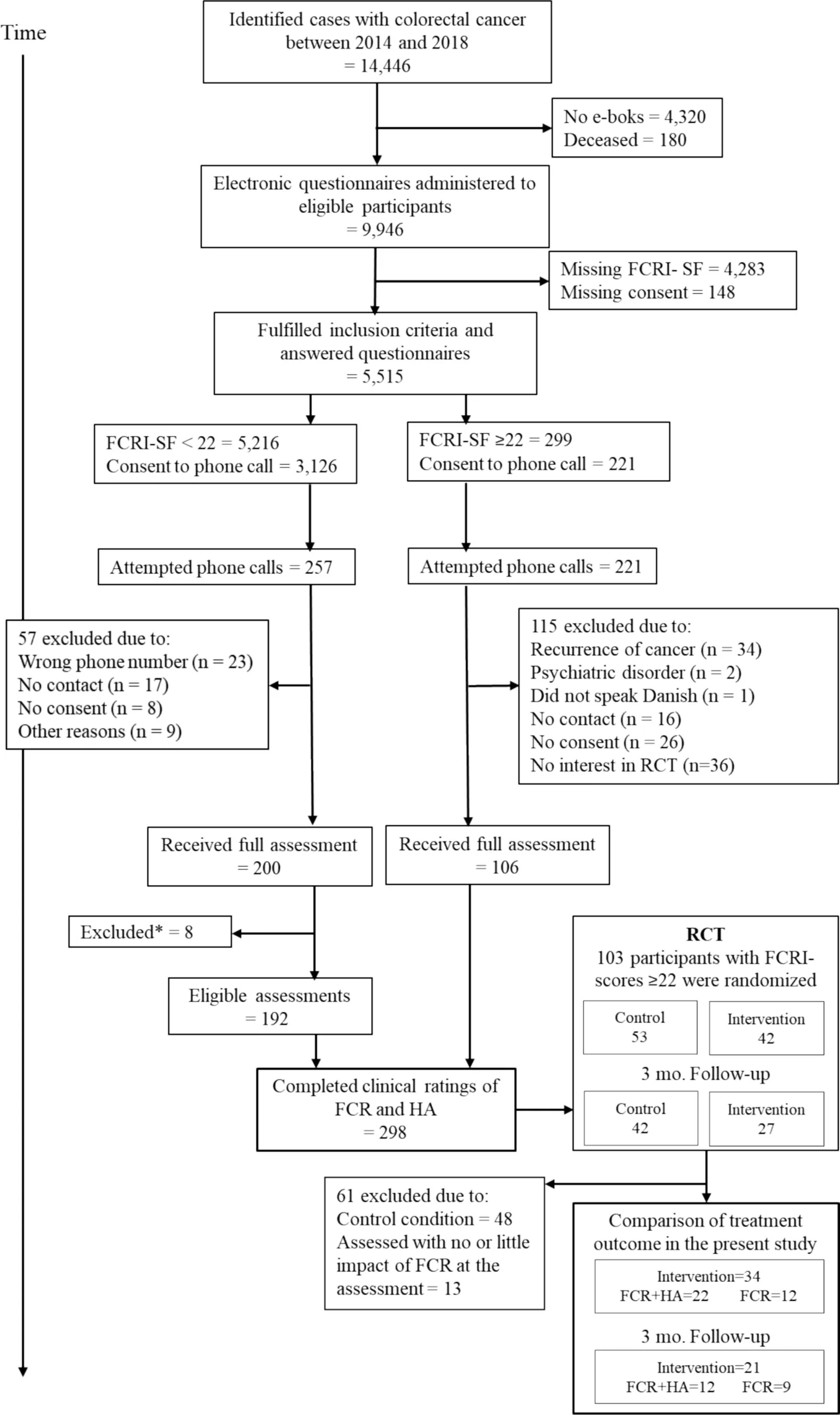
Flowchart of inclusion. FCRI-SF, Fear of Cancer Recurrence Inventory – short form; FCR, fear of cancer recurrence; RCT, randomized controlled trial. *Based on reversed item in the FCRI-SF designed to capture inattentive automatic responses

**Fig. 2:**
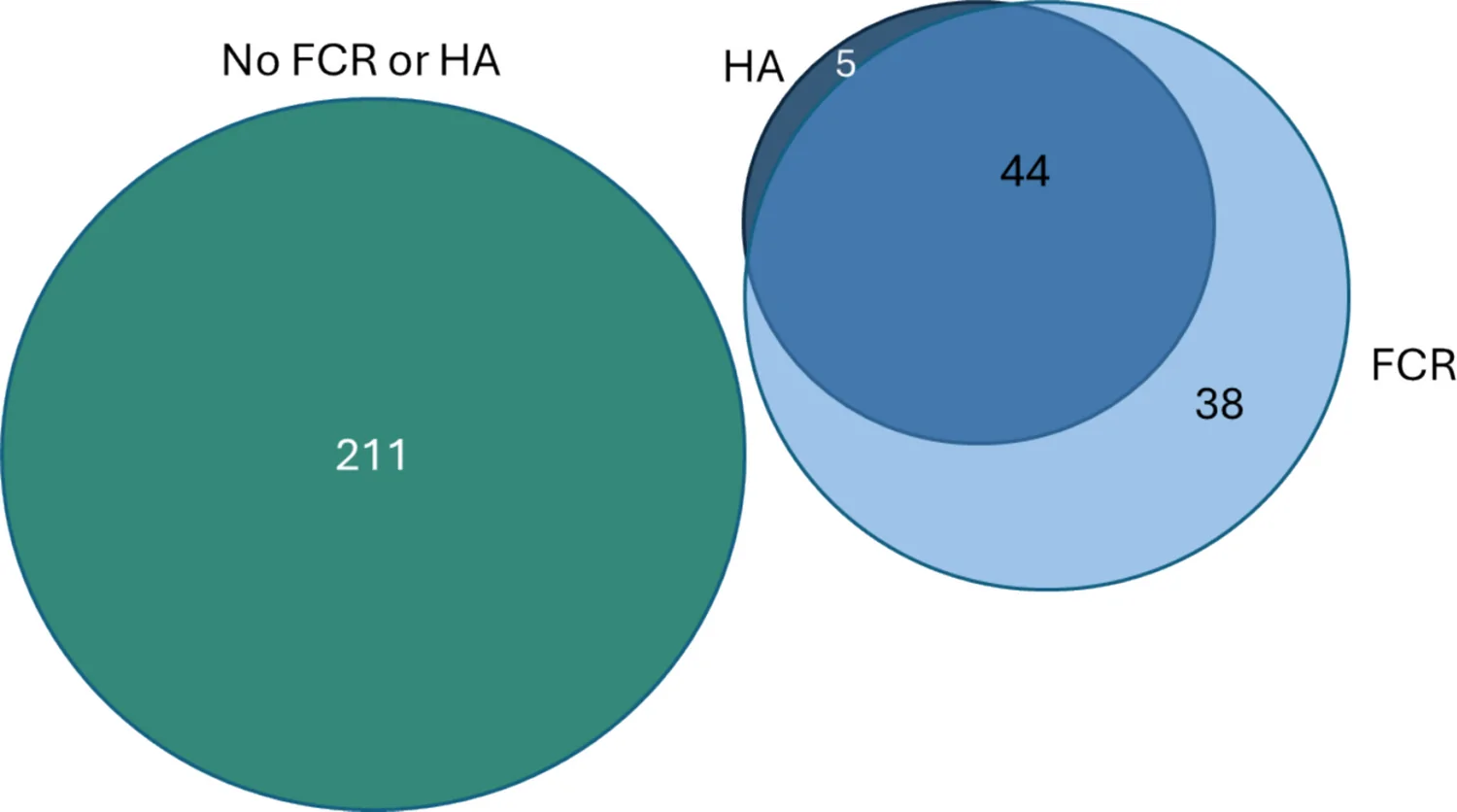
Result of clinical assessment of fear of cancer recurrence severity and health anxiety (*n* = 298). FCR, fear of cancer recurrence; HA, health anxiety

Out of the 5515 participants who answered the questionnaire, 5216 participants had an FCRI-SF score of < 22, 3126 consented to a phone call, and 200 received a diagnostic assessment. Two hundred ninety-nine participants had an FCRI-SF score ≥ 22; 221 consented to a phone call; and 106 received a diagnostic assessment (Fig. 1). Full clinical assessments were conducted with 298 participants, and participant characteristics are summarized in Table 1. Characteristics for participants with FCR only (*n* = 38) and those with both FCR and HA (*n* = 44) are also found in Table 1. The distribution of FCR and HA is summarized in Fig. 2. Due to the small number of participants with HA only (*n* = 5), comparisons in the results focused on participants with FCR only versus those with both FCR and HA.

**Table 1:** Participant characteristics

	All assessments (*N* = 298)	FCR only (*n* = 38)	Both FCR and HA (*n* = 44)	FCR only vs. both
Demographic characteristics				
Age, mean (SD)	68.7 (0.6)	61 (1.8)	62 (1.6)	*P* = 0.877^b^
Sex, % female (*n*)	49.7 (148)	68.4 (26)	68.2 (30)	*P* = 0.981^c^
Education, % (*n*)^a^				*P* = 0.960^c^
Short education (0–3 yrs)	17.4 (52)	13.2 (5)	9.1 (4)	
Medium education (3–5 yrs)	40.9 (122)	42.1 (16)	40.9 (18)	
Long education (> 5 yrs)	37.6 (112)	39.5 (15)	40.9 (18)	
Other education	3 (9)	5.3 (2)	4.6 (2)	
Missing	1 (3)	0 (0)	4.6 (2)	
Employment, % (*n*)				*P* = 0.393^c^
Employed	29.5 (88)	55.3 (21)	43.2 (19)	
Pension	67.4 (201)	44.7 (17)	54.6 (24)	
Never been employed	1 (3)	0 (0)	2.3 (1)	
Missing	2 (6)	0 (0)	0 (0)	
Married/cohabiting, % (*n*)	70.5 (210)	73.7 (28)	68.2 (30)	*P* = 0.794^c^
Children, % yes (*n*)	90.9 (271)	100 (38)	90.9 (40)	*P* = 0.057^c^
Cancer-specific characteristics				
Years since diagnosis, mean (range)	7 (4–10)	6.8 (4–9)	7.3 (4–10)	*P* = 0.149^b^
Cancer type, % (*n*)				*P* = 0.795^c^
Colon	64.1 (191)	63.2 (24)	65.9 (29)	
Rectal	35.9 (107)	36.8 (14)	34.1 (15)	
Treatment, % (*n*)				*P* = 0.936^c^
Surgery	51 (152)	42.1 (16)	38.6 (17)	
Surgery and chemo	41.6 (124)	52.6 (20)	52.3 (23)	
Surgery, chemo, and radiotherapy	7.4 (22)	5.3 (2)	6.8 (3)	
Fear of cancer recurrence				
FCRI score (0–168), mean (SD) at baseline	-	77 (3.4)	90.5 (2.8)	*P* = 0.004^b^

*FCR* fear of cancer recurrence, *HA* health anxiety, *FCRI-SF* Fear of Cancer Recurrence Inventory – short form, *yrs* year, *SD* standard deviation, *n* number

^a^Years of education after finishing 9 years of public school

^b^*t*-test

^c^Chi^2^

### Clinical characteristics from assessment

Forty-four percent of participants with both FCR and HA reported having illness-related worries before their cancer diagnosis, compared to 5.3% of participants with FCR only. For participants with FCR only, 42.1% reported that their worries were limited to colorectal cancer, while this was the case for only 2.3% of participants who also qualified for a HA diagnosis (Table 2). See supplementary for results of assessment of HA.

**Table 2:** Clinical characteristics from assessment interviews for FCR only and both FCR and HA

	FCR only (*n* = 38)	Both HA + FCR (*n* = 44)
Illness worries before cancer diagnosis, % yes (*n*)	5.3 (2)	44.2 (19)
Illness worries triggered or exacerbated by cancer, % (*n*) yes	100 (38)	90.7 (39)
Illness worries limited to, % (*n*)		
Only colorectal cancer	42.1 (16)	2.3 (1)
All types of cancer	47.4 (18)	65.1 (28)
All diseases	10.5 (4)	32.6 (14)
Number of registered physical late effects^a^, median (range)	5 (1–13)	5 (0–13)
Comorbid disorders^b^, % (*n*)	28.9 (11)	48.8 (21)

*FCR* fear of cancer recurrence, *HA* health anxiety, *n* number

^a^Late effects specific for colorectal cancer patients were counted from the following list of known late effects: abdominal pain, diarrhea/frequent loose stool, difficulty emptying bowels, involuntary passage of gas/loose stool, defecation urgency, involuntary urination, bleeding from the bladder, frequent urination, difficulty emptying the bladder, bladder pain, sexual dysfunction, peripheral neuropathy, fatigue, memory issues, difficulty concentrating

^b^Anxiety disorders, obsessive-compulsive disorders, depression

### Comparison of questionnaire data

There were no statistical differences between the two groups on the FCRI total score or any of the subscales, although participants with both FCR and HA reported higher means and medians on all measures except the *Severity* subscale. Participants with both FCR and HA did have statistically higher scores on levels of distress, anxiety, and depression as measured using the SCL subscales, but no difference was found in illness worries between the two groups (Table 3).

**Table 3:** Comparison of questionnaire data

	FCR only *N* = 38	Both FCR and HA *N* = 44	Both vs. FCR only
FCRI subscale scores			
Triggers (0–32), median sum score (IQ range)	14 (10–17)	17 (13–22)	*P* = 0.012^a^
Severity (0–36), median sum score (IQ range)	25 (23–26)	25 (23–27)	*P* = 0.138^a^
Psychological distress (0–16), median sum score (IQ range)	7.5 (5–9.5)	9 (7–12)	*P* = 0.035^a^
Coping strategies (0–36), mean sum score (SD)	18.3 (1.2)	20.5 (0.9)	*P* = 0.129^b^
Functioning impairment (0–24), mean sum score (SD)	9.6 (0.9)	10.2 (0.9)	*P* = 0.671^b^
Insight (0–12), median sum score (IQ range)	3 (3–5)	5 (3–6.5)	*P* = 0.033^a^
Reassurance (0–12), median sum score (IQ range)	2 (0–4.5)	3 (1–4)	*P* = 0.52^a^
Symptom checklist			
SCL-8 distress (0–32), mean (SD)	8 (4.5)	12 (7.1)	***P* = 0.005**^**b**^
SCL anxiety (0–16), median (IQ range)	3 (2–6)	6 (3.8)	***P* < 0.001**^**a**^
SCL depression (0–24), median (IQ range)	3 (2–5)	5 (3–10)	***P* = 0.005**^**a**^
Illness worries			
Whiteley-6-R (0–24), mean (SD)	8.1 (0.7)	11(0.9)	*P* = 0.014

Bold: significant at Bonferroni correction = 0.005. All comparisons are adjusted using Bonferroni correction

*FCR* fear of cancer recurrence, *HA* health anxiety, *FCRI* fear of cancer recurrence inventory, *SCL* symptom checklist, *IQ* interquartile, *SD* standard deviation, *n* number

^a^Mann–Whitney *U*

^b^*t*-test

### Treatment outcome

Of the participants with clinical FCR in this study, 34 received the active treatment in the RCT study—12 with FCR only and 22 with FCR and HA. These exploratory descriptive findings are reported in Table 4.

**Table 4:** Treatment outcome on the FCRI and on the Whiteley-6-R

	Baseline	3-mo. follow-up	Mean reduction
FCRI 0–168			
FCR only, mean (SD)	76.8 (19.1), *n* = 12	56.1 (25.1), *n* = 9	20.65
FCR + HA, mean (SD)	92.6 (18.3), *n* = 22	76.5 (23.4), *n* = 12	16.1
Whiteley-6-R 0–24			
FCR only, mean (SD)	8.5 (3.4), *n* = 12	4.4 (3.9), *n* = 9	4.1
FCR + HA, mean (SD)	11.5 (6.2), *n* = 22	5.9 (4.2), *n* = 12	5.6

*FCRI* fear of cancer recurrence inventory, *FCR* fear of cancer recurrence, *HA* health anxiety, *SD* standard deviation, *n* number, *mo.* months

## Discussion

The present study aimed to investigate clinical characteristics and treatment outcomes between long-term colorectal cancer survivors with FCR and HA. Overall, our findings not only indicate a substantial overlap between FCR and the HA diagnosis but also support their partial distinctiveness. Participants with both FCR and HA presented with worries predating the cancer diagnosis and more severe psychological symptoms including emotional distress, anxiety, and depression. Participants with FCR only showed a more restricted and cancer-specific worry profile. Treatment results indicate a descriptive difference in response between the two groups which generates a hypothesis and warrants more research.

Previous literature has confirmed that HA and FCR share several features—particularly high levels of preoccupation, worry, and hypervigilance toward bodily symptoms—so some argue that FCR is a form of HA [1, 9, 15, 18]. Our study suggests that when cancer-related illness worries become sufficiently pervasive and ruminative, they may meet criteria for HA as conceptualized by Fink and colleagues [15]. In our study, participants with HA frequently reported illness-related worries that predated their cancer diagnosis and included other cancer diagnoses and other illnesses. These characteristics may help clinicians recognize HA in cancer patients.

In contrast, those with FCR only typically described their concerns as focused on the risk of recurrence and were less likely to endorse generalized illness fears. This supports the conceptualization of FCR as a situationally anchored fear, in the context of a serious illness such as cancer [18, 47, 48]. In Fink and colleagues’ criteria and in DSM-5 criteria for illness anxiety disorder, it is emphasized that if a medical condition is present, the preoccupation and worry must be clearly excessive or disproportionate to fulfill HA criteria. Within FCR research, it is suggested that HA should be conceptualized differently in patients with a medical condition compared to healthy individuals [18].

Given that our sample consisted of long-term survivors (≥ 5 years post-diagnosis) with no signs of recurrence, the objective risk of relapse approximates that of the general population [49]. Therefore, in our sample, persistent and pervasive worries fit with the criteria for HA. This aligns with theoretical proposals that when illness-related fears become ruminative, chronic, and disproportionate, they meet criteria for HA even if initially triggered by cancer [21, 24]. Also, within HA research, experiences of severe illness are suggested to be a potential trigger of HA [16]. Importantly, in other cancer populations, illness worries may also present as ruminative but may still be conceptualized as FCR rather than HA, particularly when the risk of relapse is elevated, the cancer trajectory remains recent, or if the cancer can only be controlled but not cured. This emphasizes the need to consider illness trajectory when interpreting FCR and HA overlap.

Self-reported data on emotional distress, anxiety, and depression showed that participants with both clinical FCR and HA reported significantly higher levels of these three measures compared to those with FCR only. This finding may indicate greater concurrent emotional distress among individuals with comorbid HA compared to those with FCR only. Thus, it may be argued that in our sample, the group with FCR only is less impaired by their illness worries although no statistical differences were found on scores on the FCRI. In a scoping review of differences in conceptualizations of FCR, HA, worry, and uncertainty, several attributes were identified as specific for FCR, e.g., realistic fear, excessive concern about treatment adverse effects, loss of a sense of security in body, and dysfunctional processing of fear [18]. None of these characteristics was evaluated in the present study to help differentiate the characteristics of FCR from HA. In future studies, it may be relevant to investigate these characteristics for FCR only.

Regarding treatment response, firm conclusions cannot be drawn from the small sample size and the exploratory nature of the analyses. However, the results raise hypotheses regarding differential treatment response in participants with comorbid HA. Participants with both FCR and HA showed smaller reductions in FCRI scores following the therapist-guided intervention compared to those with FCR only. If treatment of FCR is less effective for patients with HA, several interesting discussion points are relevant in relation to other literature. If replicated, this pattern may suggest that interventions specifically tailored to FCR may be less well suited for individuals with co-occurring HA, hypothetically reflecting differences in underlying cognitive and metacognitive mechanisms.

A recent network analysis found that functional impairment in FCR is linked to coping strategies due to maladaptive metacognitions [50]. It may be hypothesized that individuals with FCR only in the present sample may have adaptive metacognitive beliefs, and thus less impairment and better treatment response. In contrast, severe HA is characterized by more pronounced metacognitive beliefs about illness and death (e.g., believing that worrying is helpful) [51, 52], and it may be hypothesized that control and avoidance behaviors are more complex in HA (e.g., avoiding information about illnesses). In support of this, our results generally indicate that individuals experiencing both FCR and HA represent a more complex clinical profile (e.g., fear of several illnesses, longer history of illness worries). 

Illness worries present before a cancer diagnosis may further contribute to the persistence of health-related concerns, as the cancer diagnosis may reinforce pre-existing beliefs about vulnerability and the legitimacy of worry. Therefore, the group with both FCR and HA may require more tailored treatment approaches targeting illness-related rumination and metacognitive processes rather than focusing solely on cancer-specific fears. In line with this, effective treatments of FCR focus on reframing uncertainty about one’s health, scheduling worry time, mindfulness, and managing health behaviors associated with risk for recurrence, while avoiding usual CBT methods for HA like exposure to, e.g., cancer stimuli [53]. Accordingly, TG-iConquerFear did not encourage exposure. However, given the very small sample size, these interpretations remain speculative, and future research in larger samples is needed to examine whether comorbid HA moderates treatment response in FCR interventions.

Our findings underscore the need to recognize both FCR and HA as clinically relevant phenomena. The presence of a distinguishable subgroup with FCR only supports the conceptual validity of FCR as an independent construct. Moreover, the strong phenomenological overlap with HA raises important diagnostic and treatment implications. While concerns have been raised about the potential stigmatization of cancer survivors by labeling them with a mental disorder, it is equally important to destigmatize HA as a legitimate and treatable psychological consequence of serious illness. Identifying HA in cancer survivors may facilitate appropriate referral to evidence-based interventions for HA.

### Strengths and limitations

Key strengths of this study include the use of standardized diagnostic assessment alongside validated self-report measures and population-based recruitment of long-term survivors. Although very few participants with low FCR scores were found to have FCR or HA based on clinical assessment, conducting these interviews was important to ensure that individuals with HA were not overlooked by relying solely on FCR screening for study inclusion. Another strength was the fact that the interviews were conducted by highly experienced psychologists with expertise in both FCR and HA assessment and treatment. Despite this, some limitations concern the operationalization of clinical FCR. Classification was based on psychologists’ global assessment of functional impairment and a standardized diagnostic framework (RIFD) designed to assess HA rather than predefined criteria and interviews designed for FCR. Together, the approach may limit comparability with studies using consensus-based definitions and assessments of FCR. However, the questions used from RIFD reflect the consensus-based criteria for FCR: high levels of preoccupation with symptoms, high levels of worry about recurrence that are persistent, and hypervigilance to bodily symptoms [10]. Another limitation concerns the fact that formal inter-rater reliability was not assessed as assessments were not audio-recorded. However, assessors took part in scheduled peer-supervision meetings and on-demand supervision with a medical doctor to ensure the quality of diagnostic decisions.

The sampling strategy used for clinical assessment also entailed some limitations. Participants were not selected through a purely random sampling procedure from the population of long-term cancer survivors. Instead, all participants with FCRI-SF scores ≥ 22 were invited for assessment, whereas participants with scores < 22 were selected through simple random sampling among consenting individuals. This resulted in a sample disproportionately composed of individuals with elevated FCR and at higher risk of psychological distress. Furthermore, the sampling strategy for low-scoring participants may have introduced selection bias and limited external validity as the distribution of HA and FCR may not fully represent the underlying population, thus limiting generalizability. Limitations also include the small sample size in each group which hindered statistical exploration of treatment outcome analyses as the sample sizes were just 12 in the FCR and 22 in the FCR and HA group in the treatment condition after randomization procedures in the RCT. Furthermore, of the 22, only 12 fulfilled the 3-month follow-up questionnaire due to drop out. From the paper describing the full RCT results, we know that loss to follow-up was not associated with FCR level, age, or sex and that the missingness is random [30]. Still, the sample size and missing data severely limited the conclusions that can be drawn from the comparison of the treatment outcomes. Additionally, the very small number of participants with HA only excluded direct comparisons of FCR and HA.

### Conclusions and perspectives

In conclusion, our findings indicate that FCR and HA are distinguishable, yet highly overlapping, conditions among long-term colorectal cancer survivors. Comorbid FCR and HA are associated with more severe emotional distress. These patients reported illness-related worries that predated their cancer diagnosis and that included other cancer diagnoses and other illnesses. These characteristics may help clinicians recognize when to screen their patients for HA. Treatment outcomes raised hypotheses about the need for clinical approaches aimed at comorbid HA, although the latter conclusion is based on a very small sample. Further investigation of their overlap and divergence will be essential for developing more precise conceptual frameworks, referral to effective interventions for HA when relevant, and potential tailoring of TG-iConquerFear for patients with both.

## Supplementary information

Below is the link to the electronic supplementary material.

ESM 1 (DOCX 31.6 KB)

## Data Availability

The dataset generated during this study is available from the corresponding author upon request.
