# Supplementary material for: Overlap and distinction between fear of cancer recurrence and health anxiety in long-term colorectal cancer survivors
Source: J Cancer Surviv. 2026 Jun 18;20(Suppl 2):2225–36. doi: 10.1007/s11764-026-02058-6 (PMC13744555; doi:10.1007/s11764-026-02058-6)
Supplement: Supplementary file 1 [file 11764_2026_2058_MOESM1_ESM.docx]

**Supplementary Material**

Textbox S1

**Research criteria for health anxiety as proposed by Fink et al. (2004):**

A. Obsessive rumination with intrusive thoughts, ideas, or fears of harboring an illness that cannot be stopped or can be stopped only with great difficulty.
B. One (or more) of the following five symptoms:
1. Presence of a, b, or both
   a) Worrying about or preoccupation with fears of harboring a severe physical disease or the idea that disease will be contracted in the future or preoccupation with other health concerns .
   b) Attention to and intense awareness of bodily functions, physical sensations, physiological reactions, or minor bodily problems that are misinterpreted as serious disease.
   2. Suggestibility or autosuggestibility; if the patient hears or reads about an illness, he or she is inclined to fear that he or she has the same disease.
   3. Excessive fascination with medical information.
   4. Unrealistic fear of being infected or contaminated by something touched or eaten or by a person met.
   5. Fear of taking prescribed medication.
C. If a medical condition is present, the patient’s reaction clearly exceeds what would be expected from the medical condition alone.
D. The symptoms are not better explained by another psychiatric disorder.
E. The symptoms are present for most of the time for at least 2 weeks.

Specify whether the disorder is severe or mild-severe: at least one of the symptoms in criteria A and B is severely disturbing or significantly interferes with everyday activities; mild: all others.

Table S1. Result of clinical assessment of illness worries in FCR only and both FCR and health anxiety

| **Illness worry/behavior** | FCR only (n=38) | Both HA + FCR (n=43) |
| --- | --- | --- |
| Illness ruminations, % (n) | | |
| No | 26.3 (10) | 0 (0) |
| Mild to moderate | 73.7 (28) | 44.1 (19) |
| Severe | 0 (0) | 55.9 (24) |
| Preoccupation with bodily symptoms, % (n) | | |
| No | 42.1 (16) | 14 (6) |
| Light to moderate | 57.9 (22) | 41.9 (18) |
| Severe | 0 (0) | 44.2 (19) |
| Preoccupation with health information, % (n) | | |
| No | 71.1 (27) | 44.2 (19) |
| Light to moderate | 26.3 (10) | 34.9 (15) |
| Severe | 2.6 (1) | 20.9 (9) |
| Suggestibility, % (n) | | |
| No | 60.5 (23) | 32.6 (14) |
| Light to moderate | 29 (11) | 30.2 (13) |
| Severe | 10.5 (4) | 37.2 (16) |
| Fear of contamination, % (n) | | |
| No | 97.4 (37) | 72.1 (31) |
| Light to moderate | 2.6 (1) | 18.6 (8) |
| Severe | 0 (0) | 9.3 (4) |
| Reassurance from doctor, % (n) | | |
| Accept doctor's reassurance | 63.2 (24) | 37.2 (16) |
| Unable to accept at one or more occasions | 26.3 (10) | 45.5 (20) |
| Unable to accept most of the time | 10.5 (4) | 16.3 (7) |
| Has consistently been unable to accept | 0 (0) | 0 (0) |
| Fear of taking medication, % (n) | | |
| No increased level of fear | 89.5 (34) | 81.4 (35) |
| Moderate increased level of fear | 10.5 (4) | 18.6 (8) |
| High level of fear | 0 (0) | 0 (0) |

FCR=fear of cancer recurrence; HA=health anxiety
